# Seq2Topt: a sequence-based deep learning predictor of enzyme optimal temperature

**DOI:** 10.1093/bib/bbaf114

**Published:** 2025-03-13

**Authors:** Sizhe Qiu, Bozhen Hu, Jing Zhao, Weiren Xu, Aidong Yang

**Affiliations:** Department of Engineering Science, University of Oxford, Parks Road, OX1 3PJ, Oxford, United Kingdom; Artificial Intelligence Division, School of Engineering, Westlake University, 310030, Hangzhou, China; Zhejiang University, 310058, Hangzhou, China; State Key Laboratory of Biocatalysis and Enzyme Engineering, Hubei Collaborative Innovation Center for Green Transformation of Bio-Resources, Hubei Key Laboratory of Industrial Biotechnology, School of Life Sciences, Hubei University, 430062, Wuhan, China; Tianjin Institute of Pharmaceutical Research Co. Ltd, Tianjin Binhai New Area, 300301, Tianjin, China; Tianjin Institute of Pharmaceutical Research Co. Ltd, Tianjin Binhai New Area, 300301, Tianjin, China; Department of Engineering Science, University of Oxford, Parks Road, OX1 3PJ, Oxford, United Kingdom

**Keywords:** enzyme optimal temperature, thermophilic proteins, sequence-based prediction, deep learning, attention mechanism

## Abstract

An accurate deep learning predictor is needed for enzyme optimal temperature (${T}_{opt}$), which quantitatively describes how temperature affects the enzyme catalytic activity. In comparison with existing models, a new model developed in this study, Seq2Topt, reached a superior accuracy on ${T}_{opt}$ prediction just using protein sequences (RMSE = 12.26°C and R2 = 0.57), and could capture key protein regions for enzyme ${T}_{opt}$ with multi-head attention on residues. Through case studies on thermophilic enzyme selection and predicting enzyme ${T}_{opt}$ shifts caused by point mutations, Seq2Topt was demonstrated as a promising computational tool for enzyme mining and *in-silico* enzyme design. Additionally, accurate deep learning predictors of enzyme optimal pH (Seq2pHopt, RMSE = 0.88 and R2 = 0.42) and melting temperature (Seq2Tm, RMSE = 7.57 °C and R2 = 0.64) were developed based on the model architecture of Seq2Topt, suggesting that the development of Seq2Topt could potentially give rise to a useful prediction platform of enzymes.

## Introduction

Temperature is an important influencing factor of enzyme catalysis [1], and researchers, especially of enzyme mining or engineering, want to quantitatively characterize the thermophilicity of enzymes, that is the enzyme optimal temperature (${T}_{opt}$). Given the large gap of enzyme ${T}_{opt}$ in databases (e.g. BRENDA [2]) [3] and the high cost of enzyme assays [4], using machine learning (ML) models to predict enzyme ${T}_{opt}$ has the potential to yield an optimal solution.

Most existing ML models of enzyme ${T}_{opt}$ are specific to certain enzyme classes, such as Zhang and Ge 2011’s model for xylanases [5]. Those models were usually developed on a small dataset specific to one class of enzymes, and the feature generation was mostly statistical descriptors of protein sequences (e.g. amino acid composition or dipeptide composition) [5–7]. Though some enzyme specific predictors had good accuracy (e.g. Chu *et al.* 2016’s model for beta-agarases [7] or Yan and Wu 2019’s model for beta-glucosidases (BGLs) [8]), their restricted scope limited their applications. Therefore, a general predictor of ${T}_{opt}$, regardless of enzyme classes, is needed by researchers who want to perform enzyme mining from massive sequencing data or computer aided engineering of enzymes.

Regarding ${T}_{opt}$ prediction for general enzymes, there only exist three tools, TOMER [3, 9], Preoptem [10], and DeepET [11]. TOMER, an ensemble model, can accurately predict ${T}_{opt}$ with a R2 score of 0.94, but it requires the optimal growth temperature (OGT) of the organism as an extra input other than the protein sequence. Also, the feature importance analysis of TOMER showed that the OGT contributed ~50% of its predictive power [3], which might cause prediction biases for the same enzymes expressed in different microorganisms. The strong reliance of TOMER on the OGT makes it inconvenient to use, because the OGT values are not accessible in many scenarios without organismal information (e.g. enzyme mining from metagenomics or cell-free enzyme catalysis). In contrast, Preoptem, a deep learning model using one-hot encoding and convolutional neural network (CNN), can predict ${T}_{opt}$ just from protein sequences. However, its prediction accuracy is not high (R2 = 0.36) and it cannot provide feature importance interpretation. The most recently published model of enzyme ${T}_{opt}$ was DeepET, which was based on transfer learning of OGT values. Although DeepET achieved a relatively low prediction error on its test set (RMSE = 12.2°C), it was still, to some extent, dependent on the OGT [11]. In conclusion, there lacks an accurate predictive model of enzyme ${T}_{opt}$ just using protein sequences.

With the aim to enhance the prediction accuracy of enzyme ${T}_{opt}$ just from protein sequences, this study used pre-trained protein language models (PLMs), multi-head attention mechanism, and residual dense neural networks to construct a deep learning model, Seq2Topt, with good accuracy. Also, Seq2Topt allows the interpretation of attention weights on protein residues, which helps to decipher the key sequence information for enzyme ${T}_{opt}$. Through case studies on the selection of thermophilic enzymes and predicting ${T}_{opt}$ shifts caused by point mutations, this work demonstrated that Seq2Topt could function as a powerful computational tool to aid enzyme mining and engineering.

## Materials and Methods

### Datasets

For ${T}_{opt}$, the dataset (*n* = 2917) was obtained from the GitHub repository of TOMER [9], which was originally obtained from BRENDA database [2]. 10% of the dataset was randomly split as the holdout test set (random seed = 0), and the remaining 90% of the dataset was used as the training set. The holdout test set (https://github.com/SizheQiu/Seq2Topt/tree/main/data/Topt/test.csv) was used in the model comparison of TOMER, Preoptem, DeepET, and Seq2Topt. To mediate the imbalance of ${T}_{opt}$ in the training dataset, oversampling was performed to double entries at high ${T}_{opt}$ (≥80 °C) by randomly duplicating existing entries at those value ranges (see SI, Fig. S1). The training and test datasets of melting temperature (${T}_m$) (T ≥ 50°C) were obtained from the GitHub repository of DeepTM [12]. DeepTM is a sequence based deep learning model of protein ${T}_m$. The training and test datasets of ${T}_m$ had 6240 and 1550 entries, respectively. The training, validation and test datasets of optimal pH ($p{H}_{opt}$) were obtained from the Zenodo repository of EpHod [13]. EpHod is a sequence based deep learning model of enzyme $p{H}_{opt}$. The training, validation, and test datasets of $p{H}_{opt}$ had 7124, 760, and 1971 entries, respectively. DeepTM, and EpHod were used in performance benchmarking for enzyme ${T}_m$ and $p{H}_{opt}$ prediction, respectively.

### Construction of the deep learning model

**Figure 1:**
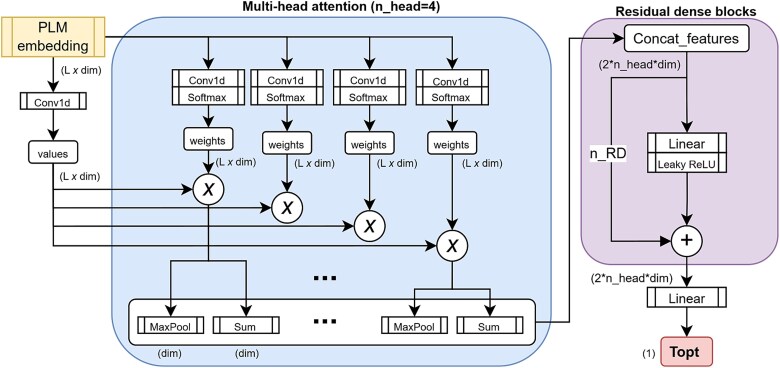
The model architecture of Seq2Topt. L: protein sequence length; dim: embedding dimension size; Conv1d: 1-D convolutional layer; ⊗: element-wise multiplication; n_head: the number of heads in multi-head attention; RD: residual dense block, a dense layer with residual connection; n_RD: the number of RDs, n_RD=4; ${T}_{opt}$: optimal temperature.

Seq2Topt consisted of PLM embedding (ESM-2 [14] or ProGen2 [15]), multi-head attention (n_head = 4) and 4 residual dense blocks (Fig. 1). First, the protein sequence embeddings ($r\in{R}^{L\ast \mathit{\dim}},L: sequence\ length,\mathit{\dim}= dimension\ size\ of\ PLM\ embeddings$) were computed by the esm2_t6_8M_UR50D model (https://github.com/facebookresearch/esm) or progen2-small (https://huggingface.co/hugohrban/progen2-small). The dimension sizes ($\mathit{\dim}$) of esm2_t6_8M_UR50D and progen2-small are 320 and 1024, respectively. The embeddings ($r$) were passed to a CNN to generate the values ($v\in{R}^{L\ast \mathit{\dim}}$), and a CNN and softmax to generate the weights ($w\in{R}^{L\ast \mathit{\dim}}$). For CNNs in this model, 3 different sliding window sizes (window size = 3, 5, 7) were experimented (see SI, Fig. S2). Then, element-wise products of values and weights were computed as the attention weighted features (${x}_{att}\in{R}^{\mathit{\dim}\ast \mathit{\dim}}$). In the multi-head attention process, the attention weighted features were computed for 4 times, and all MaxPools (${x^k}_{max}\in{R}^{1\ast \mathit{\dim}},k: index\ of\ attention\ head$) and Sums (${x^k}_{sum}\in{R}^{1\ast \mathit{\dim}},k: index\ of\ attention\ head$) of attention weighted features were concatenated as the inputs (${x}_{concat}\in{R}^{2\ast n\_ head\ast \mathit{\dim}}$) for residual dense blocks. Each residual dense block consisted of a linear layer, an activation layer, and an addition operator. The activation function used in each residual dense block was Leaky ReLU [16], which can mitigate the issue of vanishing gradients with negative input values. Also, Leaky ReLU is considered more effective than ReLU for regression tasks [17, 18]. The concatenated feature was passed through 4 residual dense blocks and a linear layer to regress for the target value.

### Deep learning model training

For the training process, batch training was used (batch size = 32) for the efficiency and generalizability of the deep learning neural network. Adam optimization algorithm [19] was used to update neural network weights iteratively. The loss function was mean squared error (MSE). The initial learning rate was 0.0005, and the learning rate decayed by 50% for every 10 epochs to prevent overfitting. Before model training started, 10% of the training set was randomly split out as the validation set (random seed = 0), and target values were rescaled as $\frac{T_{opt}}{120^{{}^{\circ}}C}$, $\frac{T_m}{100^{{}^{\circ}}C}$, and $\frac{p{H}_{opt}}{14}$. During the training process, the prediction accuracy of the model was evaluated with root MSE (RMSE), mean average error (MAE), and r-squared (R2) (see SI, Supplementary methods section S1.2). For details of software and hardware, please see section S1.1 of the supplementary information.

### Interpretation of residue attention weights

To investigate how enzyme ${T}_{opt}$ was predicted from the amino acid sequence, the average attention weights on residues (${w}_{avg}\in{R}^{L\ast 1}$) were computed by averaging the weights ($w\in{R}^{L\ast \mathit{\dim}}$) across the embedding feature dimension (dim), and across 4 attention heads. Then, the average residue attention weights (${w}_{avg}$) were mapped to the protein sequence, together with annotated active and binding sites obtained from the uniprot database [20]. The spatial distribution of high attention weights and active/binding sites could assist in revealing the hidden key sequence information influencing enzyme thermoactivity.

## Results

### The superior performance of Seq2Topt

**Figure 2:**
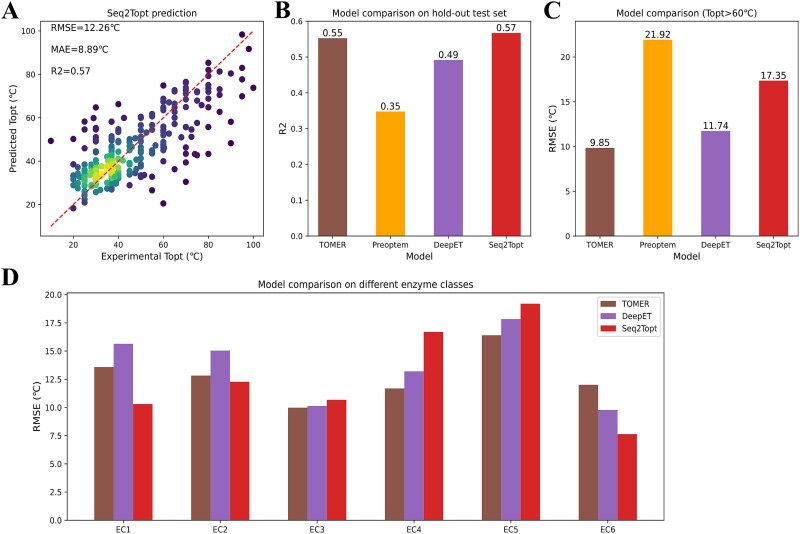
The assessment of the model performance. (a) Experimental and predicted ${T}_{opt}$ by Seq2Topt (RMSE = 12.26°C, MAE = 8.89°C, R2 = 0.57). (b) ${T}_{opt}$ prediction accuracy comparison on the same hold-out test set of TOMER, Preoptem, DeepET, and Seq2Topt. (c) ${T}_{opt}$ prediction accuracy comparison at the high value range (${T}_{opt}>{60}^{{}^{\circ}}C$) of TOMER, Preoptem, DeepET, and Seq2Topt. (d) ${T}_{opt}$ prediction accuracy comparison of TOMER, DeepET, and Seq2Topt for different enzyme classes (EC 1–6).

With the optimal PLM (ESM-2) and sliding window size of CNN (window size = 3) (see SI, Fig. S2), the training process reduced the RMSE and MAE to 12.26°C and 8.89°C, and enhanced R2 from around 0.25 to 0.57 (Fig. 2a, Fig. S3). For model comparison on the hold-out test set (*n* = 291), Seq2Topt outcompeted Preoptem and DeepET, and reached a slightly higher R2 score than TOMER (Fig. 2b). For TOMER, the prediction accuracy can be largely decreased by the random shuffling of OGT values (see SI, Fig. S4), which are not needed in ${T}_{opt}$ prediction by Seq2Topt. Regarding the high temperature value range (${T}_{opt}>{60}^{{}^{\circ}}C$), the prediction error of Seq2Topt was lower than that of Preoptem, but higher than those of DeepET and TOMER (Fig. 2c). With respect to different enzyme classes, Seq2Topt had lower prediction errors for oxidoreductases (EC1), transferases (EC2), and ligases (EC6), close prediction errors to DeepET and TOMER for hydrolases (EC3) and isomerases (EC5), a higher prediction error for lyases (EC4) (Fig. 2d). Overall, the accuracy assessment on ${T}_{opt}$ prediction exhibited the superior performance of Seq2Topt.

### Using Seq2Topt to estimate the thermophilicity of organisms and enzymes

**Figure 3:**
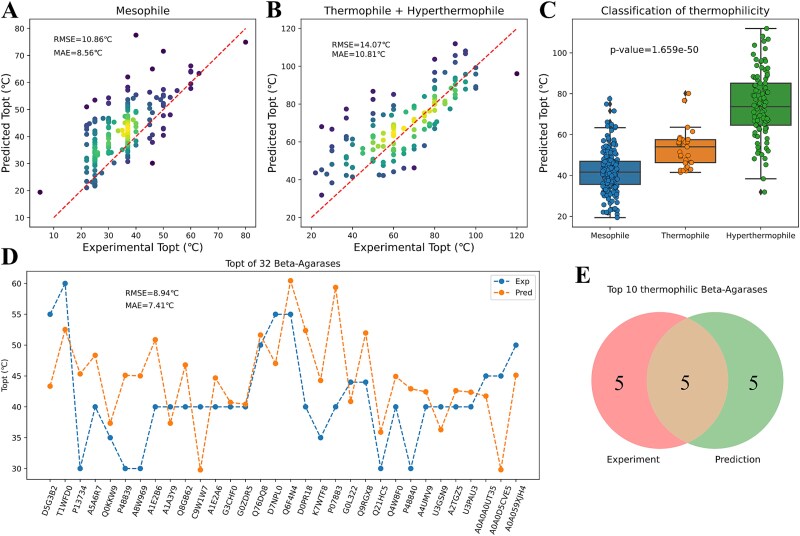
The performance of Seq2Topt on microorganisms and enzymes of different thermophilicities. (a) Experimental and predicted ${T}_{opt}$ values of enzymes from mesophilic microorganisms (RMSE = 10.86°C and MAE = 8.56°C). (b) Experimental and predicted ${T}_{opt}$ values of enzymes from thermophilic and hyperthermophilic microorganisms (RMSE = 14.07 °C and MAE = 10.81°C). (c) The distributions of predicted ${T}_{opt}$ values of enzymes from mesophilic, thermophilic, and hyperthermophilic microorganisms (*P*-value<.001). (d) Experimental and predicted ${T}_{opt}$ values of 32 beta-agarases (RMSE = 8.94 °C and MAE = 7.41 °C). (e) The Venn diagram of top 10 thermophilic beta-agarases determined by experiments and predicted by Seq2Topt.

328 enzymes from representative mesophilic, thermophilic, and hyperthermophilic microorganisms (see SI, Table S1) were selected to examine the prediction performance of Seq2Topt on microorganisms and enzymes of different thermophilicities. First, the comparison between experimental and predicted ${T}_{opt}$ values showed that Seq2Topt had good accuracy on enzymes from both mesophiles (RMSE = 10.86°C, MAE = 8.56°C) and thermophiles/hyperthermophiles (RMSE = 14.07 °C, MAE = 10.81 °C; Fig. 3a and b). The significant differential distributions of predicted ${T}_{opt}$ values of enzymes from mesophiles and thermophiles/hyperthermophiles demonstrated that Seq2Topt could classify enzymes and microorganisms of different thermophilicities (Fig. 3c).

Next, Seq2Topt was used to select thermophilic beta-agarases with the experimental data obtained from Chu *et al.*, 2016 [7]. The overall prediction error of Seq2Topt on 32 beta-agarases was relatively low (Fig. 3d), in comparison to the prediction error of Preoptem on the holdout test set (section 3.1). The 50% overlap between predicted and experimental top 10 thermophilic beta-agarases suggested that Seq2Topt, though trained on a dataset of general enzymes instead of a restricted scope, could identify thermophilic enzymes based on protein sequences (Fig. 3e).

### Deciphering the key sequence information for enzyme thermoactivity

**Figure 4:**
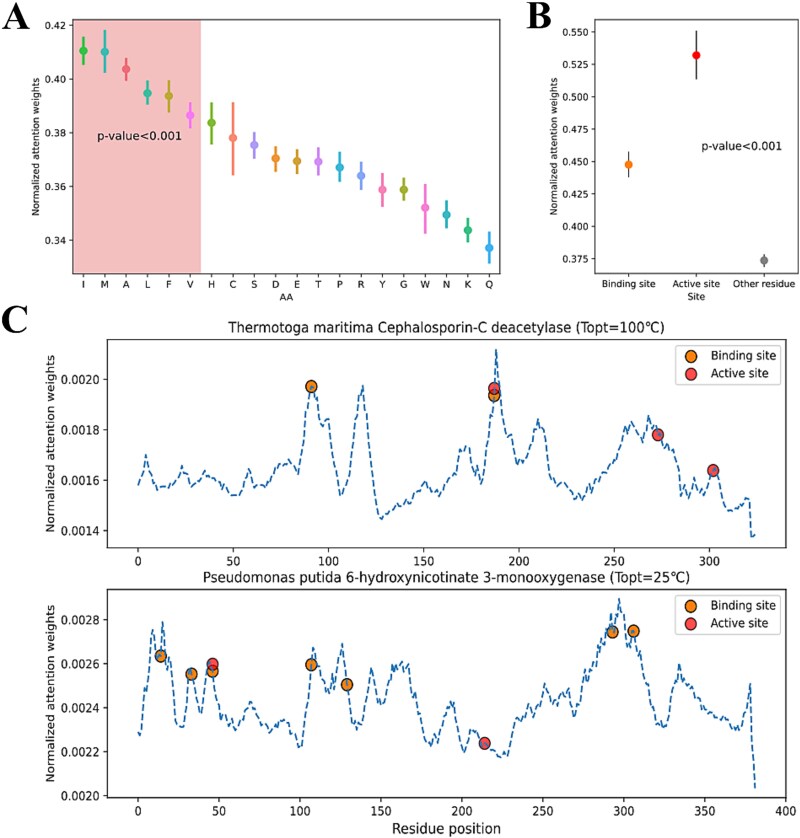
The analysis of residue attention weights. (a) The distributions of residue attention weights on 20 essential amino acids. Boxed area: 6 amino acids with significantly higher attention weights than other amino acids (*P*-value<.001). AA: amino acid. (b) The distributions of residue attention weights on active sites, binding sites, and other residues. (c) Representative examples of residue attention weights and spatial distributions of active and binding sites: *T. maritima* Cephalosporin-C deacetylase (Uniprot ID: Q9WXT2) and *P. putida* 6-hydroxynicotinate 3-monooxygenase (Uniprot ID: Q88FY2). Dashed curve: normalized attention weights; Dots: binding and active sites.

To investigate how residue attention weights of Seq2Topt capture important sequence information, this study compared the distributions of attention weights on different residues. The distributions of residue attention weights on 20 essential amino acids showed that isoleucine (I), methionine (M), alanine (A), leucine (L), phenylalanine (F), valine (V) had significantly higher weights than other amino acids (Fig. 4a). Among those 6 amino acids, I, L, and V, three branched-chain amino acids, have been reported to be related to enzyme thermoactivity and thermostability [21]. Also, the attention weights on active/binding sites were found to be significantly higher than weights on other residues (Fig. 4b), indicating that residue attention weights of Seq2Topt could capture important residues for enzyme catalytic activity. Taking *Thermotoga maritima* Cephalosporin-C deacetylase (${T}_{opt}={100}^{{}^{\circ}}C$) and *Pseudomonas putida* 6-hydroxynicotinate 3-monooxygenase (${T}_{opt}={25}^{{}^{\circ}}C$) as representative examples, most active and binding sites were located at high peaks of attention weights (Fig. 4c). In short, multi-head attention in Seq2Topt provided good interpretability by capturing key sequence information, i.e. important amino acids, active/binding sites.

### Prediction of the shift of enzyme optimal temperature caused by point mutations

**Figure 5:**
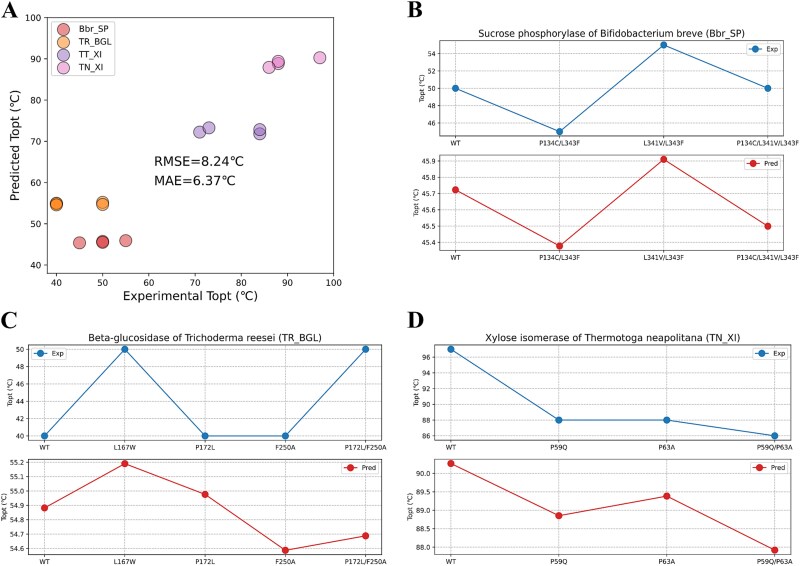
The prediction of enzyme optimal temperature shifts caused by mutations. (a) Experimental and predicted ${T}_{opt}$ values of WTs and mutants of 4 different enzymes (RMSE = 8.24°C, MAE = 6.37°C). (b) Experimental and predicted ${T}_{opt}$ values of the WT and mutants of the SP of Bbr (Bbr_SP). (c) Experimental and predicted ${T}_{opt}$ values of the WT and mutants of the BGL of TR (TR_BGL). (d) Experimental and predicted ${T}_{opt}$ values of the WT and mutants of the XI of TN (TN_XI). HC: *Heyndrickxia coagulans.*

This study used Seq2Topt to predict ${T}_{opt}$ values of wild-types (WTs) and mutants for xylose isomerases (XIs) of *Thermoanaerobacterium thermosulfurigenes* (TT) and *Thermotoga neapolitana* (TN) [22], BGL of *Trichoderma reesei* (TR) [23], and sucrose phosphorylase (SP) of *Bifidobacterium breve* (Bbr) [24] (see SI, Table S2). The experimental data of those enzymes was not included in the training set of Seq2Topt. The prediction accuracy of ${T}_{opt}$ values of WT and mutated enzymes was examined, and RMSE, MAE scores were 8.24°C and 6.37°C, respectively (Fig. 5a). The XIs of TT (TT_XI) were not included in further analysis, due to low prediction accuracy (see SI, Fig. S5). For the SP of *B. breve* (Bbr_SP), Seq2Topt qualitatively predicted that P134C/L343F and L341V/L343F could decrease and increase the ${T}_{opt}$, respectively (Fig. 5b). For the BGL of TR (TR_BGL), Seq2Topt identified that the mutation of L167W could enhance the ${T}_{opt}$, but failed to predict the increase of ${T}_{opt}$ caused by P172L/F250A (Fig. 5c). For the XI of TN (TN_XI), Seq2Topt successfully predicted that P59Q and P63A mutations could decrease the ${T}_{opt}$, and the combination of those two point mutations could result in a larger decrease of the ${T}_{opt}$ than single point mutations (Fig. 5d). To sum up, Seq2Topt could quantitatively account for the shift of ${T}_{opt}$ caused by point mutations, although the numerical variations in predicted ${T}_{opt}$ values of WTs and mutants were relatively smaller than those observed in experimental measurements.

### Seq2pHopt and Seq2Tm: use protein sequences to predict enzyme optimal pH and melting temperature

**Figure 6:**
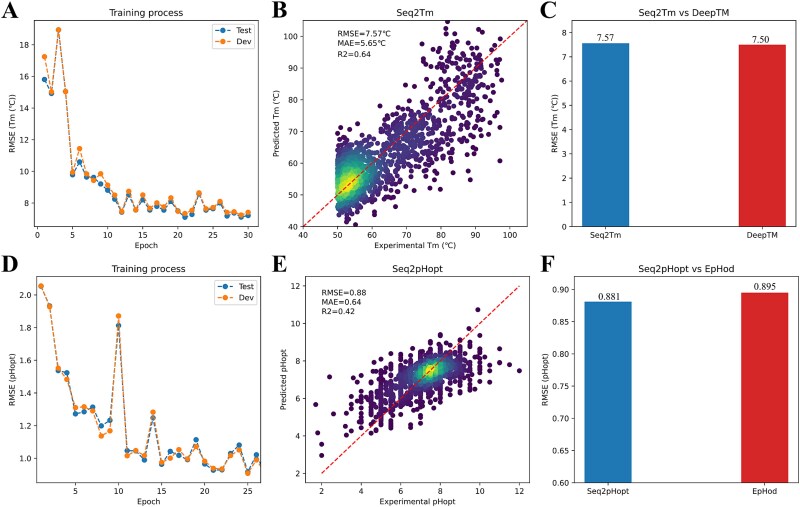
The assessment of prediction accuracy for Seq2Tm and Seq2pHopt. (a) The RMSE scores of ${T}_m$ prediction during the training process. (b) Experimental and predicted ${T}_m$ by Seq2Tm (RMSE = 7.57°C, MAE = 5.65°C, and R2 = 0.64). (c) The comparison of RMSE scores of ${T}_m$ prediction by Seq2Tm and DeepTM (RMSE of Seq2Tm = 7.57°C and RMSE of DeepTM = 7.5°C). (d) The RMSE scores of $p{H}_{opt}$ prediction during the training process. (e) Experimental and predicted $p{H}_{opt}$ by Seq2pHopt (RMSE = 0.88, MAE = 0.64, and R2 = 0.42). (f) The comparison of RMSE scores of $p{H}_{opt}$ prediction by Seq2pHopt and EpHod (RMSE of Seq2pHopt = 0.88 and RMSE of EpHod = 0.89).

The model architecture of Seq2Topt (Fig. 1) was used to construct predictive models of enzyme optimal pH ($p{H}_{opt}$) and melting temperature (${T}_m$). This study developed Seq2pHopt for $p{H}_{opt}$ and Seq2Tm for ${T}_m$ with the same CNN sliding window size and PLM as Seq2Topt (section 3.1), and both of them used the protein sequence as the only input. In the training process, the RMSE scores of Seq2Tm and Seq2pHopt were reduced from around 16 °C to 7.57 °C and from 2 to 0.88, respectively (Fig. 6 a and d). For MAE and R2 scores in the training process, please see SI, Figs S6 and S7. Seq2Tm and Seq2pHopt both achieved good accuracy on the test sets used by DeepTM [12] and EpHod [13], which are best models of ${T}_m$ and $p{H}_{opt}$ released before August, 2024 (Fig. 6b and e). In comparison to DeepTM that requires both the protein sequence and OGT as inputs, Seq2Tm could reach a closely good prediction accuracy without using the OGT (Fig. 6c). For $p{H}_{opt}$ prediction, Seq2pHopt outperformed EpHod with a lower RMSE score (Fig. 6f). Generally speaking, Seq2Tm and Seq2pHopt had superior prediction accuracy on ${T}_m$ and $p{H}_{opt}$, respectively.

## Discussion

The gap of experimental data and expensive cost of enzyme ${T}_{opt}$ demand an accurate and easy-to-use predictive model, and this study managed to tackle this challenging task and developed Seq2Topt, a deep learning model that can predict enzyme ${T}_{opt}$ just from protein sequences. Three main elements of Seq2Topt were PLM embedding of protein sequences, multi-head attention, and residual dense neural networks (Fig. 1). Compared with conventional feature extraction methods of protein sequences, such as one-hot encoding [10] or k-mer based dictionary embedding [25, 26], PLM embedding of protein sequences has the advantage of being able to learn the information of structures and functions hidden in sequences [14, 15]. Due to the relatively small dataset size (*n* = 2917), one-hot or k-mer based encoding might fail to include certain amino acid subsequences, rendering some protein sequences unencodable. In contrast to single-head attention, multi-head attention could improve the prediction accuracy by focusing on different parts of protein sequences simultaneously [27], which has been demonstrated by the higher accuracy of Seq2pHopt than EpHod that uses single-head attention [13] (Fig. 6f). Also, the attention weights of Seq2Topt effectually captured important sequence information, such as branched-chain amino acids with high feature importance to enzyme ${T}_{opt}$ (section 3.3). In addition, the use of residue dense neural networks instead of multiple linear layers could effectively reduce the vanishing and exploding gradient issues in deep neural networks [28]. Also, oversampling on entries at the high temperature value range, to some extent, compensated for the imbalanced distribution of enzyme ${T}_{opt}$ values in the dataset (section 2.1). As a result, Seq2Topt outperformed other existing enzyme ${T}_{opt}$ predictors with RMSE = 12.26 °C and R2 = 0.57 (Fig. 2a).

Case studies of selecting thermophilic beta-agarases (section 3.2) and predicting enzyme ${T}_{opt}$ shifts caused by point mutations (section 3.4) manifested that Seq2Topt could be applied to enzyme mining and computational design of enzymes via fast screening the effect of mutations. Expectedly, the combination of Seq2Topt and generative deep learning might lead to predictor-guided generator optimization [29] of enzymes, enabling automatic enzyme design. Furthermore, all three accurate predictive models of enzyme ${T}_{opt}$, $p{H}_{opt}$, and ${T}_m$ can potentially improve the performance of condition dependent enzyme ${k}_{cat}$ prediction (e.g. DLTKcat [26] or MPEK [30]) by informing the catalytic optimum.

Despite the achievement of Seq2Topt, there still exist some limitations for Seq2Topt, which hinder the improvement of prediction accuracy. First, its accuracy in the high temperature value range is relatively low (Fig. 2), and the main reason lies in the imbalance of the dataset. Also, the size of the dataset used to develop Seq2Topt is much smaller than those of other deep learning models for proteins, such as the dataset of EpHod containing 9855 enzymes [13]. One possible solution is to append the dataset of enzyme ${T}_{opt}$ by curating more entries from enzyme databases (e.g. BRENDA [2]) or conducting high-throughput enzyme assays, especially for the high temperature value range. Another shortcoming of Seq2Topt is that it cannot account for the impact of environmental factors on the thermoactivity of enzymes [31], such as pH [32], enzyme concentrations in assays [33], and salt concentrations [34–36]. Including metadata of curated experimental measurements might resolve this shortcoming, but the lack of enzyme assay metadata in commonly used enzyme databases impedes this approach.

In conclusion, Seq2Topt is an accurate and easy to use (the only input needed is the protein sequence) deep learning predictor of enzyme ${T}_{opt}$, in spite of some limitations discussed above. As envisaged, Seq2Topt can potentially accelerate enzyme discovery for desired properties from ‘biological dark matter’ and enzyme engineering with *in-silico* design, and might give rise to a powerful prediction platform of enzymes.

**Key Points**

- Seq2Topt can accurately predict enzyme optimal temperature values just from protein sequences.
- Seq2Topt can predict the shift of enzyme optimal temperature caused by point mutations.
- Residue attention weights of Seq2Topt can reveal important sequence regions for enzyme thermoactivity.
- The architecture of Seq2Topt can be used to build predictors of other enzyme properties (e.g. optimal pH).

## Supplementary Material

SI_TOPT_rev_bbaf114

## Data Availability

The code and data are openly available at https://github.com/SizheQiu/Seq2Topt.
