# Supplementary material for: Seq2Topt: a sequence-based deep learning predictor of enzyme optimal temperature
Source: Brief Bioinform. 2025 Mar 13;26(2):bbaf114. doi: 10.1093/bib/bbaf114 (PMC11904407; doi:10.1093/bib/bbaf114)
Supplement: SI_TOPT_rev_bbaf114 [file si_topt_rev_bbaf114.docx]

**Seq2Topt**: a sequence-based deep learning predictor of enzyme optimal temperature

## Authorship

Sizhe Qiu^1‡^, Bozhen Hu^2,3‡^, Jing Zhao^4,5^, Weiren Xu^5^, Aidong Yang^1^*

^1^Department of Engineering Science, University of Oxford, OX1 3PJ, United Kingdom

^2^AI Division, School of Engineering, Westlake University, Hangzhou 310030, China

^3^Zhejiang University, Hangzhou 310058, China

^4^State Key Laboratory of Biocatalysis and Enzyme Engineering, Hubei Collaborative Innovation Center for Green Transformation of Bio-Resources, Hubei Key Laboratory of Industrial Biotechnology, School of Life Sciences, Hubei University, Wuhan 430062, China

^5^Tianjin Institute of Pharmaceutical Research Co., Ltd., Tianjin 300301, China

^‡^Equal contribution

*Corresponding author: [aidong.yang@eng.ox.ac.uk](mailto:aidong.yang@eng.ox.ac.uk) (A. Yang)

##

## 1. Supplementary methods

### 1.1 Software and code availability

All scripts were written in python. The deep learning model was implemented using PyTorch v1.7.1. The computer used in this work was a Dell Latitude Laptop with intel core i7 CPU. The model was trained with GPU RTX8000 provided by Advanced Research Computing (ARC) service in the University of Oxford [[1]](https://paperpile.com/c/rih2ej/McUo). Figures were edited using InkScape (<https://inkscape.org/>). The code and data used to generate results of this paper are available at <https://github.com/SizheQiu/Seq2Topt>.

### 1.2 Evaluation metrics

To quantitatively assess the prediction accuracy, R2 (Eq. S1), RMSE (Eq. S2), and MAE (Eq. S3) were computed for each test.

$$R^{2}=\frac{\sum_{i=1}^{n} (y_{ie}-y_{ip})^{2}}{\sum_{i=1}^{n} (y_{ie}-\bar{y})^{2}} (Eq. S1)$$

$$RMSE=\sqrt{\frac{1}{n}\sum_{i=1}^{n} (y_{ie}-y_{ip})^{2}} (Eq. S2)$$

$MAE = \frac{1}{n}\sum_{i=1}^{n} \left| y_{ie}-y_{ip} \right|(Eq. S3)$

## 2. Supplementary figures


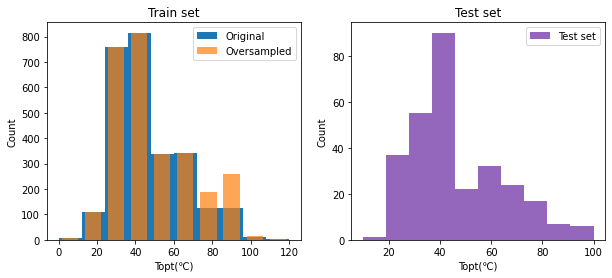


Figure S1. The distribution of enzyme $T_{opt}$ values in training and test sets.


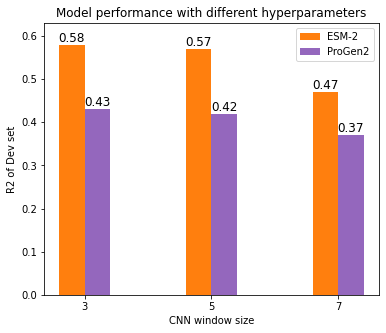


Figure S2. Hyperparameter optimization for $T_{opt}$ prediction on different CNN window sizes (window size=3,5,7) and two different protein language models (ESM-2 and ProGen2). The optimal window size is 3, and the optimal protein language model is ESM-2.


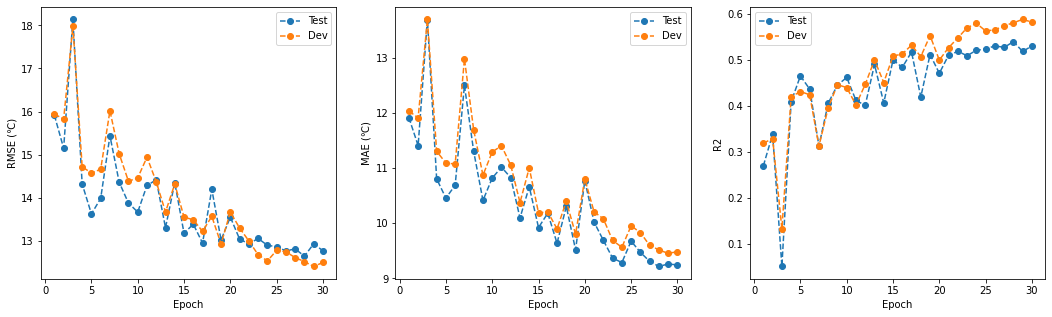


Figure S3. The training process of Seq2Topt in 30 epochs.


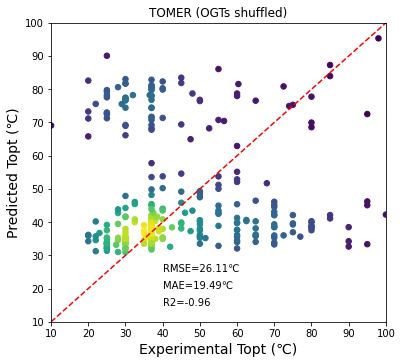


Figure S4. The RMSE, MAE and R2 scores of TOMER when OGT values are shuffled.


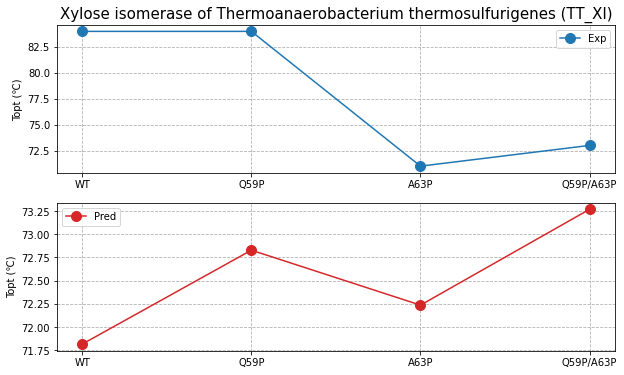


Figure S5. Experimental and predicted $T_{opt}$ values of the wild-type and mutants of the xylose isomerase of *Thermoanaerobacterium thermosulfurigenes* (TN_XI).


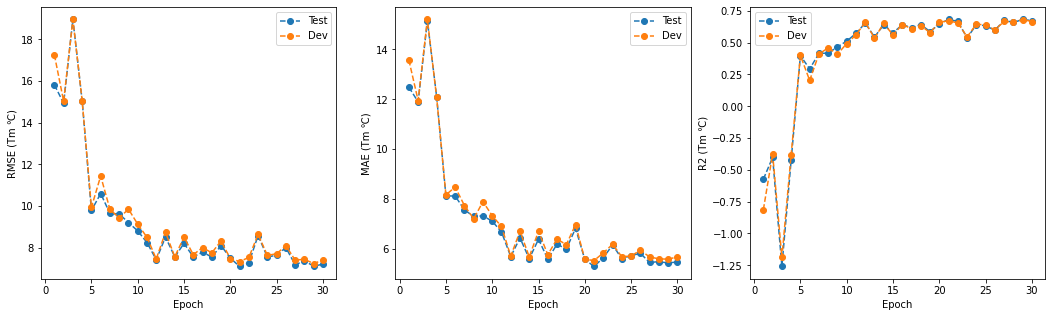


Figure S6. The training process of Seq2Tm in 30 epochs. Tm: melting temperature.


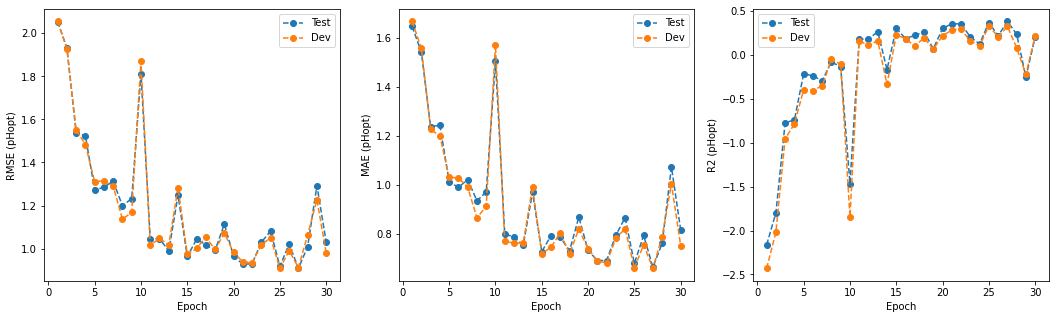


Figure S7. The training process of Seq2pHopt in 30 epochs. pHopt: optimal pH.


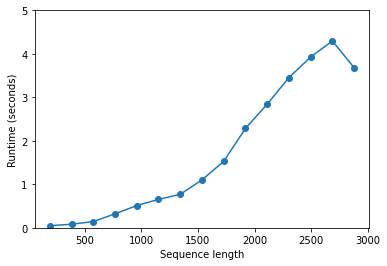


Figure S8. The change of runtime of Seq2Topt on CPU (11th Gen Intel(R) Core(TM) i7-1185G7 @ 3.00GHz, RAM 16GB) with respect to protein sequence lengths.

##

## 3. Supplementary tables

Table S1. Selected microorganisms for the estimation of thermophilicity

| Organism | Class | OGT (℃) |
| --- | --- | --- |
| Escherichia coli | Mesophile | 36 |
| Bacillus subtilis | Mesophile | 30 |
| Staphylococcus aureus | Mesophile | 37 |
| Geobacillus stearothermophilus | Thermophile | 54 |
| Geobacillus thermoleovorans | Thermophile | 60 |
| Geobacillus thermodenitrificans | Thermophile | 57 |
| Pyrococcus furiosus | Hyperthermophile | 96 |
| Thermotoga maritima | Hyperthermophile | 75 |
| Pyrococcus abyssi | Hyperthermophile | 90 |
| Aquifex aeolicus | Hyperthermophile | 80 |

Table S2. Information of wild-type and mutated enzymes used in case studies.

| Enzyme | Organism | Mutation | Topt (℃) | Reference |
| --- | --- | --- | --- | --- |
| Beta-glucosidase | Trichoderma reesei | WT | 40 | [[2]](https://paperpile.com/c/rih2ej/0uKAT) |
|  |  | L167W | 50 |  |
|  |  | P172L | 40 |  |
|  |  | F250A | 40 |  |
|  |  | P172L/F250A | 50 |  |
| Xylose isomerase | Thermotoga neapolitana | WT | 97 | [[3]](https://paperpile.com/c/rih2ej/aeJSD) |
|  |  | P59Q | 88 |  |
|  |  | P63A | 88 |  |
|  |  | P59Q/P63A | 86 |  |
| Xylose isomerase | Thermoanaerobacterium thermosulfurigenes | WT | 84 | [[3]](https://paperpile.com/c/rih2ej/aeJSD) |
|  |  | Q59P | 84 |  |
|  |  | A63P | 71 |  |
|  |  | Q59P/A63P | 73 |  |
| Sucrose phosphorylase | Bifidobacterium breve | WT | 50 | [[4]](https://paperpile.com/c/rih2ej/C87r) |
|  |  | P134C/L343F | 45 |  |
|  |  | L341V/L343F | 55 |  |
|  |  | P134C/L341V/L343F | 50 |  |

* values were extracted from plots using WebPlotDigitizer (<https://github.com/automeris-io/WebPlotDigitizer>) [[5]](https://paperpile.com/c/rih2ej/5QeY).

## Reference

[1. Richards A. University of Oxford Advanced Research Computing. 2015;](http://paperpile.com/b/rih2ej/McUo)

[2. Lee H-L, Chang C-K, Jeng W-Y, et al. Mutations in the substrate entrance region of β-glucosidase from Trichoderma reesei improve enzyme activity and thermostability. Protein Eng. Des. Sel. 2012; 25:733–740](http://paperpile.com/b/rih2ej/0uKAT)

[3. Sriprapundh D, Vieille C, Zeikus JG. Molecular determinants of xylose isomerase thermal stability and activity: analysis of thermozymes by site-directed mutagenesis. Protein Eng. 2000; 13:259–265](http://paperpile.com/b/rih2ej/aeJSD)

[4. Zhou Y, Ke F, Chen L, et al. Enhancing regioselectivity of sucrose phosphorylase by loop engineering for glycosylation of L-ascorbic acid. Appl. Microbiol. Biotechnol. 2022; 106:4575–4586](http://paperpile.com/b/rih2ej/C87r)

[5. Drevon D, Fursa SR, Malcolm AL. Intercoder Reliability and Validity of WebPlotDigitizer in Extracting Graphed Data. Behav. Modif. 2017; 41:323–339](http://paperpile.com/b/rih2ej/5QeY)
